# Obstructive sleep apnoea-related respiratory events and desaturation severity are associated with the cardiac response

**DOI:** 10.1183/23120541.00121-2022

**Published:** 2022-10-24

**Authors:** Salla Hietakoste, Tuomas Karhu, Saara Sillanmäki, Raquel Bailón, Thomas Penzel, Juha Töyräs, Timo Leppänen, Sami Myllymaa, Samu Kainulainen

**Affiliations:** 1 Dept of Applied Physics, University of Eastern Finland, Kuopio, Finland; 2 Diagnostic Imaging Center, Kuopio University Hospital, Kuopio, Finland; 3 Aragón Institute of Engineering Research, University of Zaragoza, Zaragoza, Spain; 4 Centro de Investigación Biomédica en Red en Bioingeniería, Biomateriales y Nanomedicina, Zaragoza, Spain; 5 Interdisciplinary Sleep Medicine Center, Charité-Universitätsmedizin Berlin, Berlin, Germany; 6 Dept of Biology, Saratov State University, Saratov, Russian Federation; 7 Science Service Center, Kuopio University Hospital, Kuopio, Finland; 8 School of Information Technology and Electrical Engineering, The University of Queensland, Brisbane, Australia

## Abstract

**Background:**

Obstructive sleep apnoea (OSA) causes, among other things, intermittent blood oxygen desaturations, increasing the sympathetic tone. Yet the effect of desaturations on heart rate variability (HRV), a simple and noninvasive method for assessing sympathovagal balance, has not been comprehensively studied. We aimed to study whether desaturation severity affects the immediate HRV.

**Methods:**

We retrospectively analysed the electrocardiography signals in 5-min segments (n=39 132) recorded during clinical polysomnographies of 642 patients with suspected OSA. HRV parameters were calculated for each segment. The segments were pooled into severity groups based on the desaturation severity (*i.e.* the integrated area under the blood oxygen saturation curve) and the respiratory event rate within the segment. Covariate-adjusted regression analyses were performed to investigate possible confounding effects.

**Results:**

With increasing respiratory event rate, the normalised high-frequency band power (HF_NU_) decreased from 0.517 to 0.364 (p<0.01), the normalised low-frequency band power (LF_NU_) increased from 0.483 to 0.636 (p<0.01) and the mean RR interval decreased from 915 to 869 ms (p<0.01). Similarly, with increasing desaturation severity, the HF_NU_ decreased from 0.499 to 0.364 (p<0.01), the LF_NU_ increased from 0.501 to 0.636 (p<0.01) and the mean RR interval decreased from 952 to 854 ms (p<0.01). Desaturation severity-related findings were confirmed by considering the confounding factors in the regression analyses.

**Conclusion:**

The short-term HRV response differs based on the desaturation severity and the respiratory event rate in patients with suspected OSA. Therefore, a more detailed analysis of HRV and desaturation characteristics could enhance OSA severity estimation.

## Abstract 2

**Higher short-term HRV is related to more severe oxygen desaturations and a higher rate of respiratory events. Considering HRV and desaturation characteristics in the diagnosis of OSA could be useful when assessing the cardiac consequences of OSA.** https://bit.ly/3yZSYTR

## Introduction

Obstructive sleep apnoea (OSA) is one of the most prevalent sleep disorders increasing the risk for cardiovascular diseases and other severe sequelae [1–3]. In OSA, nocturnal respiratory events, *i.e.* apnoeas and hypopnoeas, cause physiological consequences such as oxygen desaturations and oscillations of the heart rate [4]; therefore, they also affect heart rate variability (HRV) [5, 6]. HRV is an effective, noninvasive measure to evaluate the state of the autonomic nervous system, especially the activities of its sympathetic and parasympathetic branches regulating the vital functions of the body [7]. Yet, electrocardiogram (ECG) usage is very limited in the diagnostics of OSA, although it is routinely recorded during polysomnography (PSG) [8].

Together with neuro-cardiac interactions, the sympathetic nervous system (SNS) and parasympathetic nervous system (PNS) regulate the functioning of the heart and, thus, HRV [7]. Normally, stress increases SNS activity, which is associated with increased oxidative stress, systemic inflammation, decreased long-term (≥24 h) HRV and a higher risk for coronary artery disease [9, 10]. Conversely, the PNS dominates the sympathovagal balance during rest, elevating long-term HRV [10]. In OSA, the frequent respiratory events shift the sympathovagal balance towards SNS dominance by causing intermittent hypoxaemia, hypercapnia, intrathoracic pressure changes and recurrent arousals [3]. Furthermore, OSA patients have reduced long-term HRV [5, 11] but the events also lead to increased ultra-short-term time-domain HRV [12]. Long-term HRV cannot be used to assess the immediate physiological responses to respiratory events and hypoxaemia, although decreased long-term HRV is generally associated with poor health [10, 13].

Currently, the severity assessment of OSA is predominantly based on the average number of respiratory events per hour of sleep, *i.e.* the apnoea-hypopnoea index (AHI), determined from PSG [8]. The AHI does not consider the severity of individual respiratory events or their physiological consequences. Therefore, novel computational parameters using integrated area under the blood oxygen saturation curve have been developed [14, 15] and have a stronger association with the consequences of OSA than the AHI [15–18]. Longer respiratory events cause more severe desaturations and increased heart rate after the event [4, 19] and are associated with higher ultra-short-term (<5 min) HRV [12]. Nonetheless, the effect of desaturations on HRV has not been comprehensively studied in patients with OSA. Because HRV parameters can be easily derived from ECG measurements, we hypothesised that they could be used more extensively to assess the state of the autonomic nervous system in OSA patients. Therefore, in the future they could potentially evaluate the risk for cardiovascular diseases alongside conventional diagnostic parameters.

In this study, our main hypothesis was that, in the short term (5 min), more severe desaturations are associated with higher time-domain HRV, increased low-frequency band power and blunted high-frequency band power in frequency-domain HRV. In addition, we hypothesised that the severity of desaturations affects the short-term HRV more strongly than the rate of respiratory events. Using a large clinical population of patients with a suspicion of OSA, we aimed to investigate whether the severity of desaturations and the rate of respiratory events affect time- and frequency-domain HRV.

## Methods

### PSG data

**FIGURE 1:**
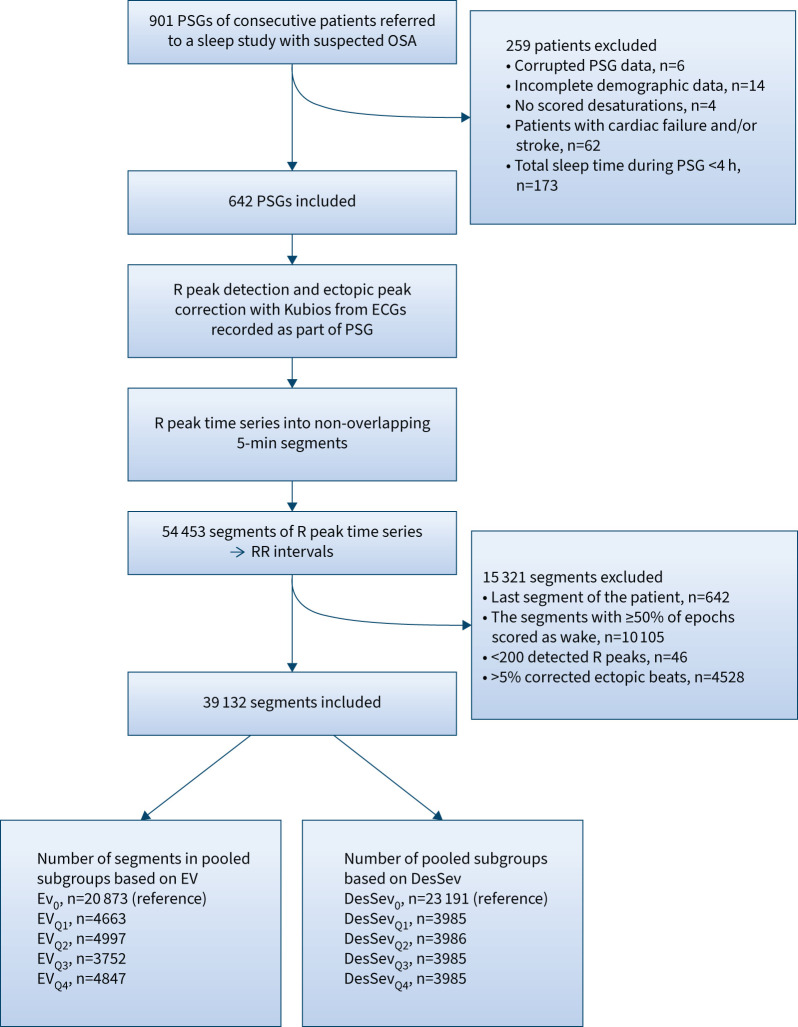
Inclusion and exclusion criteria for the patient and 5-min segment selection. PSG: polysomnography; OSA: obstructive sleep apnoea; ECG: electrocardiogram; Ev: event rate; Ev_0_=0 events per 5 min, Ev_Q1_=1 event per 5 min<Ev_Q2_≤3 events per 5 min<Ev_Q3_≤5 events per 5 min<Ev_Q4_; DesSev: desaturation severity; DesSev_0_=0%, DesSev_Q1_≤0.305%<DesSev_Q2_≤0.906%<DesSev_Q3_≤2.463%<DesSev_Q4_.

We retrospectively studied the type I PSGs of 901 consecutive patients referred to a sleep study owing to clinical suspicion of OSA. The PSGs were recorded at the Princess Alexandra Hospital (Brisbane, Australia) between 2015 and 2017 using the Grael Acquisition System (Compumedics, Abbotsford, Australia). The recordings were manually scored by experienced sleep technicians in compliance with the American Academy of Sleep Medicine 2012 scoring criteria [8]. A respiratory event was scored as an apnoea if the respiratory airflow decreased by ≥90% from the baseline for ≥10 s. An event was scored as a hypopnoea if the respiratory airflow decreased ≥30% from the baseline for ≥10 s and there was arousal or a ≥3% drop in blood oxygen saturation related to the respiratory event. The Institutional Human Research Ethics Committee of Princess Alexandra Hospital approved the data collection and re-use (HREC/16/QPAH/021 and LNR/2019/QMS/54313). After patient exclusion (criteria described in figure 1), we included a total of 642 patients in this study (table 1).

**TABLE 1:** Demographic characteristics of the study population

**Characteristics**	
**Patients (male %)**	642 (54.4%)
**Age (years)**	53.4 (42.9–63.1)
**BMI (kg·m^−2^)**	34.4 (28.9–40.0)
**AHI (events·h^−1^)**	16.9 (7.7–36.0)
**OSA**	
None (AHI<5)	107 (16.7%)
Mild (5≤AHI<15)	185 (28.8%)
Moderate (15≤AHI<30)	163 (25.4%)
Severe (AHI≥30)	187 (29.1%)
**ODI_3%_ (events·h^−1^)**	13.6 (4.6–33.1)
**Total sleep time (min)**	330.3 (287.5–370.0)
**All 5-min segments**	39 132
**Events per 5-min segment**	
Ev_0_	20 873 (53.3%)
Ev_Q1_	4663 (11.9%)
Ev_Q2_	4997 (12.8%)
Ev_Q3_	3752 (9.6%)
Ev_Q4_	4847 (12.4%)
**Desaturation severity**	
DesSev_0_	23 191 (59.3%)
DesSev_Q1_	3985 (10.2%)
DesSev_Q2_	3986 (10.2%)
DesSev_Q3_	3985 (10.2%)
DesSev_Q4_	3985 (10.2%)
**Comorbidities**	
Atrial arrhythmia	56 (8.7%)
COPD	58 (9.0%)
Diabetes mellitus, type 1	3 (0.5%)
Diabetes mellitus, type 2	115 (17.9%)
Hypothyroidism	65 (10.1%)
Hypertension	239 (37.2%)

Data are presented as median (interquartile range) for continuous variables and as n (%) for discrete variables. BMI: body mass index; AHI: apnoea-hypopnoea index; OSA: obstructive sleep apnoea; ODI_3%_: oxygen desaturation index based on American Academy of Sleep Medicine 2012 scoring criteria (desaturation ≥3%); Ev_0_: reference group with 0 respiratory events starting during the 5-min segment; Ev_Q1–4_: severity groups pooled based on the number of respiratory events (apnoeas/hypopnoeas) starting during the 5-min segment (Ev_Q1_=1<Ev_Q2_≤3<Ev_Q3_≤5<Ev_Q4_); DesSev_0_: reference group with desaturation severity of 0 during the 5-min segment; DesSev_Q1–4_: severity groups pooled based on the desaturation severity in the 5-min segment (0%<DesSev_Q1_≤0.305%<DesSev_Q2_≤0.906%<DesSev_Q3_≤2.463%<DesSev_Q4_); COPD: chronic obstructive pulmonary disease.

### HRV analysis

ECGs were recorded during the PSGs using lead II [8] with a sampling frequency of 256 Hz. We detected the R peaks from the ECGs using the Kubios HRV Premium 3.4.1 software (Kubios Oy, Kuopio, Finland) with the default settings [20]. The Kubios detects R peaks with the algorithm based on the Pan–Tompkins method [21] using the amplitude threshold and expected time between adjacent R peaks as the decision rules. They both are adaptively adjusted after each detected R peak. To improve the time resolution, the R wave is interpolated to 2000 Hz before extracting the time of the R peak. The software also uses automatic correction for artefacts due to ectopic peaks and missed peak detections; detected artefact peaks are replaced using cubic spline interpolation. We divided the resulting R peak time series into non-overlapping 5-min segments during sleep (n=54 453). The exclusion criteria for the segments are described in figure 1.

Further, we divided the remaining 5-min segments (n=39 132) into severity groups based on the respiratory event rate (Ev), including any event starting during the segment, *i.e.* apnoeas and hypopnoeas. We considered the segments having zero apnoeas and hypopnoeas as the reference group (Ev_0_, n=20 873). The remaining segments (n=18 259) were divided into quartiles Ev_Q1_ to Ev_Q4_ with thresholds of 1, 3 and 5 events in the 5-min segment (Ev_Q1_=1<Ev_Q2_≤3<Ev_Q3_≤5<Ev_Q4_, table 1). A similar division was separately performed based on the severity of the desaturations starting during the segment. The severity of desaturations was assessed using the desaturation severity (DesSev) parameter that describes the severity of hypoxic load by considering the depth and duration of the individual desaturation events as the integrated area under the blood oxygen saturation curve. The DesSev was calculated by dividing the sum of individual desaturation areas by the duration of the segment (5 min) [14]. The segments having DesSev=0 (DesSev_0_, n=23 191) were considered as the reference group. The remaining segments (n=15 941) were pooled into quartiles DesSev_Q1_ to DesSev_Q4_ with thresholds of 0.305%, 0.906% and 2.463% (0% <DesSev_Q1_≤0.305%<DesSev_Q2_≤0.906%<DesSev_Q3_≤2.463%<DesSev_Q4_, table 1).

Next, we computed the values of short-term (5 min) time- and frequency-domain HRV parameters for each 5-min segment. The time-domain HRV parameters consisted of the mean RR interval, the standard deviation of the RR intervals from which artefacts had been corrected (SDNN), the root mean square of the successive differences (RMSSD) and the proportion of adjacent RR intervals differing by more than 50 ms (pRR_50_) [7]. Before the frequency-domain analysis, the 5-min RR interval segments were resampled by cubic spline interpolation and detrended by the smoothness priors method with λ=500 [22]. Then, we estimated the power spectral densities (PSDs) with Welch's method [20]. From PSDs, we calculated the frequency-domain HRV parameters: power in the high-frequency band (HF) (0.15–0.40 Hz), the low-frequency band (LF) (0.04–0.15 Hz), and their ratio (LF/HF ratio) [7]. We additionally calculated the normalised HF and LF band powers, HF_NU_=HF/(HF+LF) and LF_NU_=LF/(HF+LF), respectively [7]. The HF and HF_NU_ band powers are thought to represent PNS activity, whereas both SNS and PNS activities contribute to the LF and LF_NU_ band powers [7]. Finally, we calculated the medians of HRV parameter values separately for the Ev and DesSev groups and compared them between 1) Ev groups, 2) DesSev groups and 3) the corresponding Ev and DesSev groups.

Because the severity groups could contain numerous 5-min segments from the same patient, we assumed the groups not to be independent. Therefore, we used a Wilcoxon signed-rank test to evaluate the statistical significance of differences in cases 1), 2) and 3). Because the Wilcoxon signed-rank test conducts a pairwise comparison, we computed a total of 5000 randomly chosen permutations of the HRV parameter pairs in each of cases 1), 2) and 3). Only one HRV parameter was compared between two severity groups at a time. We defined the statistical significance as the median of the 5000 p-values and a significance level of p<0.01 was used for these analyses owing to large sample sizes and multiple testing.

To consider the effect of potential confounding factors on HRV parameters, we also performed covariate-adjusted regression analysis for the 5-min segments. DesSev was the continuous variable and sex, age, body mass index (BMI), daytime sleepiness based on the Epworth Sleepiness Scale (ESS) questionnaire, history of arrhythmias, hypertension, hypothyroidism and chronic obstructive pulmonary disease (COPD) were adjusting covariates. The HRV, statistical and regression analyses were performed with MATLAB R2018b (MathWorks Inc., Natick, MA, USA).

## Results

### Frequency-domain HRV

**FIGURE 2:**
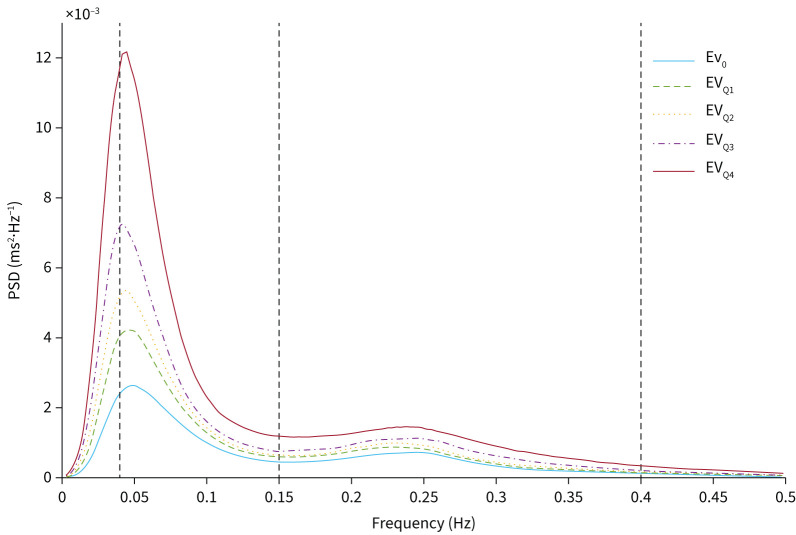
Median power spectral densities (PSDs) in the severity groups pooled based on the respiratory event rate (Ev) within the 5-min segments. Vertical dashed lines separate the frequency bands used in heart rate variability analyses: the low-frequency band (0.04–0.15 Hz) and the high-frequency band (0.15–0.40 Hz). Ev_0_=0 events per 5 min, Ev_Q1_=1 event per 5 min<Ev_Q2_≤3 events per 5 min<Ev_Q3_≤5 events per 5 min<Ev_Q4_.

The HF, LF and LF_NU_ band powers, and the LF/HF ratio increased while the HF_NU_ decreased with the increasing number of respiratory events within the 5-min segments (table 2, figure 2). All frequency-domain HRV parameter values in different Ev groups were different from the reference group Ev_0_ (p<0.001). The LF band power increased between each Ev group towards the Ev_Q4_ group (p<0.001).

**TABLE 2:** Frequency-domain HRV parameter values in the severity groups based on the Ev and the DesSev in the 5-min segments

**Severity group**	**HF (ms^2^)**	**LF (ms^2^)**	**LF/HF**	**HF_NU_**	**LF_NU_**
**Events per 5-min segment**				
Ev_0_	183.6 (57.5–523.1)	160.9 (56.7–451.1)	0.934 (0.433–2.012)	0.517 (0.332–0.698)	0.483 (0.302–0.668)
Ev_Q1_	**198.1 (57.1–585.6)**	225.9 (76.7–644.0)**	1.243 (0.584–2.504)**	0.446 (0.285–0.631)**	0.554 (0.369–0.715)**
Ev_Q2_	**207.0 (62.5–608.2)**	258.1 (98.4–708.9)**	**1.385 (0.647–2.731)**	**0.419 (0.268–0.607)**	**0.581 (0.393–0.732)**
Ev_Q3_	**236.0 (76.0–653.6)**	327.0 (129.6–866.2)**	**1.421 (0.730–2.805)**	**0.413 (0.263–0.578)**	**0.587 (0.422–0.737)**
Ev_Q4_	314.9 (99.5–841.0)**	536.3 (203.9–1292.3)**	1.745 (0.817–3.664)**	0.364 (0.214–0.550)**	0.636 (0.450–0.786)**
**Desaturation severity**				
DesSev_0_	198.5 (63.5–550.3)^##^	186.6 (64.5–525.5)^##^	1.004 (0.462–2.127)^##^	0.499 (0.320–0.684)^##^	0.501 (0.316–0.680)^##^
DesSev_Q1_	175.5 (48.0–557.1)	**213.3 (69.9–632.9)**	**1.303 (0.597–2.693)**	**0.434 (0.271–0.626)**	**0.566 (0.374–0.729)**
DesSev_Q2_	199.9 (60.5–577.7)	**229.5 (83.2–653.0)^##^**	**1.256 (0.577–2.559)^##^**	**0.443 (0.281–0.634)^##^**	**0.557 (0.366–0.719)^##^**
DesSev_Q3_	**210.6 (65.5–652.8)**	282.4 (103.9–757.3)**^,^ ^**##**^	**1.347 (0.654–2.788)**	**0.426 (0.264–0.605)**	**0.574 (0.395–0.736)**
DesSev_Q4_	287.1 (92.8–861.0)**	479.7 (173.8–1310.3)**	1.747 (0.818–3.462)**	0.364 (0.224–0.550)**	0.636 (0.450–0.776)**

The median of frequency-domain HRV parameter values was calculated from each 5-min RR interval segment. Data are presented as the median (interquartile range). Statistical significance of differences between 1) Ev groups, 2) DesSev groups and 3) the corresponding Ev and DesSev groups was assessed using the Wilcoxon signed-rank test. Bolded values denote a statistically significant (p<0.01) difference compared to the corresponding reference group.

HRV: heart rate variability; Ev: respiratory event rate; Ev_0_: reference group with 0 respiratory events starting during the 5-min segment; Ev_Q1–4_: severity groups pooled based on the number of respiratory events (apnoeas/hypopnoeas) starting during the 5-min segment (Ev_Q1_=1<Ev_Q2_≤3<Ev_Q3_≤5<Ev_Q4_); DesSev: desaturation severity; DesSev_0_: reference group with desaturation severity of 0% during the 5-min segment; DesSev_Q1–4_: severity groups pooled based on the desaturation severity during the 5-min segment (0%<DesSev_Q1_≤0.305%<DesSev_Q2_≤0.906%<DesSev_Q3_≤2.463%<DesSev_Q4_); HF: total power in the high-frequency band (0.15–0.40 Hz); LF: total power in the low-frequency band (0.04–0.15 Hz); HF_NU_: normalised power in the high-frequency band (HF/(HF+LF)); LF_NU_: normalised power in the low-frequency band (LF/(HF+LF)). **: statistically significant difference (p<0.01) compared to all other Ev or DesSev groups; ^##^: statistically significant difference (p<0.01) compared to the corresponding Ev group.

**FIGURE 3:**
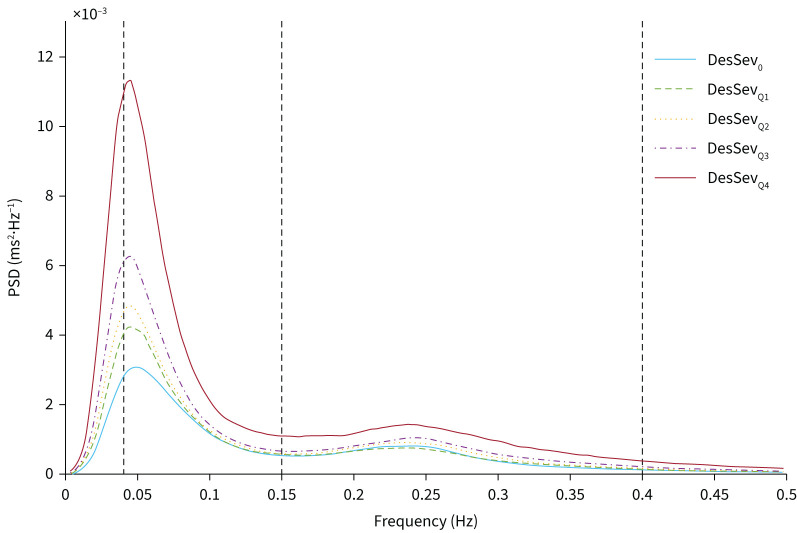
Median power spectral densities (PSDs) in the groups pooled based on the desaturation severity (DesSev) within the 5-min segments. Vertical dashed lines separate the frequency bands used in heart rate variability analyses: the low-frequency band (0.04–0.15 Hz) and the high-frequency band (0.15–0.40 Hz). DesSev_0_=0%, DesSev_Q1_≤0.305%<DesSev_Q2_≤0.906%<DesSev_Q3_≤2.463%<DesSev_Q4_.

The LF and LF_NU_ band powers increased and the HF_NU_ power decreased towards the more severe DesSev groups (table 2, figure 3). Similarly, the HF band power and the LF/HF ratio showed a trend for an increase towards the most severe DesSev group (DesSev_Q4_). LF and LF_NU_ band powers and LF/HF ratio values in all DesSev groups were higher than in the DesSev_0_ group (p<0.001). The frequency-domain HRV parameter values in the DesSev_Q4_ group were higher than all other DesSev groups (p<0.001).

All HRV parameter values in the DesSev_0_ group, except HF_NU_, were higher than in the Ev_0_ group (p<0.001, table 2). In the DesSev_Q2_ and DesSev_Q3_ groups, the LF band power was lower than in the Ev_Q2_ and Ev_Q3_ groups (p<0.001), respectively.

### Time-domain HRV

A higher Ev was associated with the shorter mean RR interval and higher other short-term time-domain HRV parameter values (table 3). Within the most severe event group, Ev_Q4_, the mean RR intervals were shorter (p<0.001) and the SDNN, RMSSD and pRR50 parameter values were greater (p<0.001) than in the reference group Ev_0_ and all other Ev groups. The increase in the SDNN values was significant (p≤0.002) between all Ev groups.

**TABLE 3:** Time-domain HRV parameter values in the severity groups based on the Ev and the DesSev in the 5-min segments

**Severity group**	**Mean RR (ms)**	**SDNN (ms)**	**RMSSD (ms)**	**pRR50 (%)**
**Events per 5-min segment**				
Ev_0_	915.3 (815.3–1033.1)	42.8 (26.6–67.9)	33.3 (18.8–59.5)	7.0 (0.8–28.0)
Ev_Q1_	913.2 (814.6–1028.4)	53.8 (33.6–84.4)**	35.2 (19.2–63.8)	7.8 (1.0–27.8)
Ev_Q2_	907.7 (807.4–1026.3)	58.9 (38.2–90.4)**	**36.5 (20.4–66.4)**	8.4 (1.5–27.8)
Ev_Q3_	**898.9 (801.4–1019.1)**	66.7 (43.3–98.0)**	40.7 (22.8–70.2)**	**9.6 (2.1–27.6)**
Ev_Q4_	868.5 (784.2–973.9)**	73.9 (49.3–104.6)**	47.4 (27.1–80.8)**	14.1 (4.1–32.3)**
**Desaturation severity**				
DesSev_0_	925.2 (824.3–1044.3)^##^	45.7 (28.3–72.1)^##^	34.7 (19.8–61.2)^##^	8.2 (1.0–29.5)^##^
DesSev_Q1_	**898.9 (797.0–1010.3)** ^##^	53.3 (31.4–85.4)**	34.1 (17.8–63.2)	**6.7 (0.8–25.6)**
DesSev_Q2_	**897.8 (801.3–1010.6)**	56.5 (35.0–88.5)**^,##^	35.8 (19.5–64.7)	8.2 (1.2–26.4)
DesSev_Q3_	878.6 (785.0–994.3)**^,##^	60.3 (39.2–91.3)**^,##^	38.5 (21.0–68.2)**	8.3 (1.5–26.7)
DesSev_Q4_	853.6 (771.4–952.5)**^,##^	75.5 (51.8–106.6)**^,##^	46.7 (25.9–82.3)**	12.2 (3.6–30.6)**^,##^

The median of time-domain HRV parameter values was calculated from each 5-min RR interval segment. Data are presented as median (interquartile range). Statistical significance of differences between 1) Ev groups, 2) DesSev groups and 3) the corresponding Ev and DesSev groups was assessed using the Wilcoxon signed-rank test. The bolded values denote a statistically significant (p<0.01) difference compared to the corresponding reference group. HRV: heart rate variability; Ev: respiratory event rate; Ev_0_: reference group with 0 respiratory events starting during the 5-min segment; Ev_Q1–4_: severity groups pooled based on the number of respiratory events (apnoeas/hypopnoeas) starting during the 5-min segment (Ev_Q1_=1<Ev_Q2_≤3<Ev_Q3_≤5<Ev_Q4_); DesSev: desaturation severity; DesSev_0_: reference group with desaturation severity of 0% during the 5-min segment; DesSev_Q1–4_: severity groups pooled based on the desaturation severity during the 5-min segment (0%<DesSev_Q1_≤0.305%<DesSev_Q2_≤0.906%<DesSev_Q3_≤2.463%<DesSev_Q4_); SDNN: standard deviation of RR intervals (artefacts corrected); RMSSD: root mean square of successive differences; pRR50: the number of adjacent RR intervals differing more than 50 ms. **: statistically significant difference (p<0.01) compared to all other Ev or DesSev groups; ^##^: statistically significant difference (p<0.01) compared to the corresponding Ev group.

Similarly, shorter mean RR intervals and higher other short-term time-domain HRV parameter values were observed with more severe desaturations (table 3). Mean RR intervals in all DesSev groups were shorter and SDNN values greater than in the reference group DesSev_0_ (p<0.001). The mean RR intervals were significantly shorter and SDNN and RMSSD values higher in the DesSev_Q3_ and DesSev_Q4_ groups than in all other DesSev groups (p<0.001).

In the reference group DesSev_0_, all time-domain HRV parameter values were greater (p<0.001) than in the Ev_0_ group (table 3). The mean RR interval and SDNN values in DesSev groups differed from the corresponding Ev groups in the majority of comparisons (p<0.001). The decrease in mean RR intervals from the DesSev_0_ to DesSev_Q4_ group was greater than that of the corresponding Ev groups.

### Desaturation characteristics in the severity groups

Within the Ev groups, the median number and depth of the desaturations within the 5-min segments increased towards the more severe Ev groups (p<0.001, table 4). However, the desaturations were the longest in the Ev_Q3_ group (p<0.001). Similarly, the median number, duration and depth of the desaturations consistently increased towards the more severe DesSev groups (p<0.001). In general, the desaturations were longer and deeper (p<0.001) within the DesSev groups compared to the corresponding Ev groups.

**TABLE 4:** Desaturation characteristics in the 5-min segments in the severity groups

**Severity group**	**n_desat_**	**Duration (s)**	**Depth (%)**	**DesSev (%)**
**Events per 5-min segment**				
Ev_0_	0 (0–0)	0 (0–0)	0 (0–0)	0 (0–0)
Ev_Q1_	1 (0–1)	12.0 (0–29.0)**	3.0 (0–3.0)**	0.062 (0–0.251)**
Ev_Q2_	2 (1–3)	27.0 (8.1–39.2)**	3.0 (2.0–4.0)**	0.335 (0.037–0.825)**
Ev_Q3_	4 (2–5)	34.8 (24.6–46.3)**	4.3 (3.3–6.7)**	1.250 (0.500–2.726)**
Ev_Q4_	7 (5–8)	30.5 (24.4–37.3)**	5.7 (4.0–9.6)**	2.662 (1.328–4.916)**
**Desaturation severity**				
DesSev_0_	0 (0–0)	0 (0–0)	0 (0–0)	0 (0–0)
DesSev_Q1_	1 (1–1)	22.1 (17.0–30.0)**^,##^	3.0 (3.0–3.0)**^,##^	0.161 (0.105–0.226)**^,##^
DesSev_Q2_	2 (2–3)	31.5 (24.2–41.0)**^,##^	3.5 (3.0–4.0)**^,##^	0.531 (0.402–0.685)**^,##^
DesSev_Q3_	4 (3–6)	34.3 (26.8–43.7)**^,##^	4.3 (3.7–5.3)**^,##^	1.504 (1.166–1.923)**^,##^
DesSev_Q4_	6 (5–8)	37.8 (30.7–47.2)**^,##^	9.0 (6.5–13.6)**^,##^	4.471 (3.305–6.948)**^,##^

Data are presented as median (interquartile range). Statistical significance of differences between 1) Ev groups, 2) DesSev groups and 3) the corresponding Ev and DesSev groups was assessed using the Wilcoxon signed-rank test. The bolded values denote a statistically significant (p<0.01) difference compared to the corresponding reference group. Ev_0_: reference group with 0 respiratory events starting during the 5-min segment; Ev_Q1–4_: severity groups pooled based on the number of respiratory events (apnoeas/hypopnoeas) starting during the 5-min segment (Ev_Q1_=1<Ev_Q2_≤3<Ev_Q3_≤5<Ev_Q4_); DesSev_0_: reference group with desaturation severity of 0% during the 5-min segment; DesSev_Q1–4_: severity groups pooled based on the desaturation severity during the 5-min segment (0%<DesSev_Q1_≤0.305%<DesSev_Q2_≤0.906%<DesSev_Q3_≤2.463%<DesSev_Q4_); n_desat_: median number of desaturations within the 5-min segments.**: statistically significant difference (p<0.01) compared to all other Ev or DesSev groups; ^##^: statistically significant difference (p<0.01) compared to the corresponding Ev group.

### Covariate-adjusted regression analysis

Covariate-adjusted regression analysis showed that even after adjustments, increasing DesSev decreased the HF_NU_ power and mean RR interval and increased the LF_NU_ power and other time-domain HRV parameters (table 5). Overall, male sex was associated with lower HF_NU_ power, higher LF_NU_ power and higher time-domain HRV parameter values. Age blunted the total PSD power and increased the mean RR intervals, and higher BMI was associated with lower LF_NU_ power, mean RR intervals and the SDNN. The ESS score was only marginally associated with HRV. Of the comorbidities, a history of arrhythmias had the greatest effect on HRV parameters, decreasing the LF_NU_ power and increasing the HF_NU_ power and time-domain HRV parameter values.

**TABLE 5:** Covariate-adjusted regression analysis to investigate the effect of covariates on selected heart rate variability parameters

	**HF_NU_**		**LF_NU_**		**Mean RR**		**SDNN**	
	**β±se**	**t-stat**	**β**±**se**	**t-stat**	**β±se**	**t-stat**	**β±se**	**t-stat**
**Sex**	−0.048**±**0.002***	−23.2	0.035**±**0.002***	19.0	0.038**±**0.002***	23.7	0.009**±**0.000***	20.0
**Age**	−0.000**±**0.000	−3.1	−0.001**±**0.000***	−17.4	0.002**±**0.000***	29.9	−0.000**±**0.000***	−6.0
**BMI**	0.001**±**0.000***	9.4	−0.002**±**0.000***	−20.3	−0.004**±**0.000***	−49.4	−0.000**±**0.000***	−17.1
**ESS**	0.001**±**0.000***	5.5	−0.000**±**0.000	−3.2	−0.000**±**0.000	−0.9	0.000**±**0.000***	4.8
**Hypothyroidism**	0.017**±**0.003***	5.0	−0.018**±**0.003***	−5.8	−0.000**±**0.003	−0.2	−0.007**±**0.001***	−8.8
**Arrhythmias**	0.090**±**0.004***	22.5	−0.073**±**0.004***	−20.7	0.073**±**0.003***	23.9	0.036**±**0.001***	39.5
**Hypertension**	0.006**±**0.002	2.8	−0.018**±**0.002***	−9.3	0.002**±**0.002	1.1	−0.007**±**0.001***	−14.7
**COPD**	−0.004**±**0.004	−1.1	−0.033**±**0.003***	−10.0	−0.080**±**0.003***	−28.0	−0.011**±**0.001***	−13.1
**DesSev**	−0.015**±**0.000***	−32.2	0.005**±**0.000***	11.8	−0.008**±**0.000***	−23.5	0.004**±**0.000***	39.7

Sex and all comorbidities are considered as categorical variables in the regression model. BMI: body mass index; ESS: Epworth Sleepiness Scale; COPD: chronic obstructive pulmonary disease; DesSev: desaturation severity; β: estimated coefficient for the regression model; t-stat: t-statistic for a test that the coefficient is zero; HF_NU_: normalised power in the high-frequency band (HF/(HF+LF)); LF_NU_: normalised power in the low-frequency band (LF/(HF+LF)); SDNN: standard deviation of RR intervals (artefacts corrected). ***: p<0.001.

## Discussion

In this study, we investigated whether the severity of the desaturation events (DesSev) and the rate of the respiratory events (Ev) affect the short-term time- and frequency-domain HRV parameters. To our knowledge, the connection between desaturation severity and HRV has not previously been studied in OSA patients. Supporting our hypothesis, we observed that the more severe desaturations were associated with decreased normalised HF band power and higher absolute and normalised LF band powers, LF/HF ratio and short-term time-domain HRV parameter values. In addition, a higher Ev was associated with higher absolute LF band power, LF/HF ratio and mean RR intervals compared to corresponding desaturation severity groups.

Previous studies show that, compared with the AHI, the severity of the nocturnal hypoxic load has a stronger association with OSA symptoms and comorbidities such as daytime sleepiness, heart failure and cardiovascular disease-related mortality [15–18]. Against our hypothesis, the higher rate of respiratory events (apnoeas and hypopnoeas) within 5-min segments was related to generally higher absolute time-domain HRV parameter values and SNS activation based on frequency-domain HRV compared to the corresponding DesSev groups (tables 2 and 3). Greater changes were also seen in HRV between reference and other groups with Ev quartiles (tables 2 and 3). This finding seems fairly counterintuitive because, generally, DesSev quartiles include more desaturations and they are significantly longer and deeper compared to the corresponding Ev quartiles (table 4). Nevertheless, a higher Ev seems to lead to stronger SNS activation (table 2). By contrast, intermittent respiratory events lead to cyclical heart rate variation [23], potentially increasing the short-term HRV, whereas longer desaturations may result in the heart rate decreasing more steadily or even stabilising at a certain level. However, other OSA-related factors could partly explain these differences. Shorter respiratory events lead to less severe desaturations [19] but increase the arousability [24, 25]. Arousals increase the heart rate and the SNS activity [26] and, therefore, they most probably contribute to our findings. In our present study, increasing both DesSev and Ev in the 5-min segments led to higher short-term HRV, which is logical because desaturations are a consequence of the respiratory events. The driving force behind these differences in HRV between Ev and DesSev groups remains unclear and more comprehensive research, including other potential factors such as sleep stages and arousals, is required.

The frequency-domain HRV analysis showed that all parameter values, except HF_NU_, increased with the increasing Ev and increasing DesSev. There was a higher relative increase in absolute LF compared with absolute HF band power towards the more severe DesSev and Ev groups, which elevated the LF/HF ratio (table 2, figures 2 and 3). In addition, the LF_NU_ band power increased and HF_NU_ power decreased towards more severe DesSev and Ev groups (table 2). These findings indicate a shift towards sympathetic dominance. Previous studies show that patients with OSA have higher SNS activity compared to healthy controls [6, 27, 28]. In addition, they demonstrate an overactive SNS compared to PNS in terms of higher LF band power and LF/HF ratio with more severe OSA, and blunted HF band power [5, 6, 29, 30]. Our present results suggest that increasing hypoxic load leads to a significantly stronger SNS activation. These findings are in line with previous studies [5, 6, 27–30] because longer respiratory events cause more severe desaturations [19]. Based on the present findings and previous literature [2, 3, 15, 31], OSA patients with more severe hypoxic load are at higher risk for cardiovascular diseases because sympathetic overdrive and severe desaturations are major risk factors for numerous other cardiovascular diseases. Long-term intermittent hypoxaemia increases the chemosensitivity of the carotid body and leads to increased SNS activity [32]. Sympathetic overdrive is also one predisposing factor for early morning cardiovascular events [29] and has been suggested to partly explain the increased propensity for arrhythmias in OSA patients [33]; the risk of arrhythmia is higher in OSA patients and is shown to significantly increase shortly after respiratory events [34, 35]. In the present study, patients with a history of arrhythmias had lower sympathetic tone than patients with no arrhythmia history (table 5) but this might have been due to possible medications stabilising heart function. Although these results may manifest the increased cardiovascular risk linked to more severe desaturations, more detailed research on respiratory events, desaturations and their effect on cardiovascular stress is warranted.

In agreement with our hypothesis, the time-domain HRV parameter values increased with the increasing Ev and DesSev. In Ev and DesSev groups, the mean RR interval decreased significantly and other time-domain parameters (SDNN, RMSSD and pRR50) increased towards the more severe Ev and DesSev groups (table 3). This finding is expected because the number, duration and depth of desaturations increased towards the more severe groups (table 4). Furthermore, longer respiratory events result in greater changes in RR intervals, higher ultra-short-term (<5 min) HRV and more severe desaturations [12, 19]. Some studies [6, 28] have also shown that more severe OSA can lead to higher long-term time-domain HRV, although OSA is commonly linked with decreased long-term HRV [5, 11] and autonomic nervous system regulation [36]. However, because we investigated the short-term HRV, these previous studies [6, 28] are not directly comparable to our present findings. Guilleminault *et al.* [23] have shown that respiratory events are accompanied by cyclical heart rate variation, and the vagally mediated respiratory sinus arrhythmia is known to contribute to *e.g* SDNN variation within short-term analyses [7]. The higher time-domain HRV parameter values could, therefore, be affected by the hyperpnoea following respiratory events. Another potential factor explaining these findings is that the body adapts to the recurrent intermittent hypoxaemia [37]. Thus, increased short-term HRV might be more harmful owing to increased beat-to-beat variation within a short time, although decreased long-term HRV generally indicates poor health [10, 13]. Based on both time- and frequency-domain HRV results, more severe desaturations and a higher Ev lead to stronger SNS activation. HRV analyses could thus be used alongside the conventional OSA severity parameters, *e.g*. the AHI, to assess the physiological consequences of OSA and their severity.

The main limitation of this study was not considering the sleep stages or arousals from sleep when analysing HRV within the 5-min segments. Transitions between sleep stages modulate HRV alongside arousals because heart rate decreases towards deeper sleep [26, 37–40] and, therefore, affected our results. We decided not to include sleep stages in our analysis because the 5-min segments consisted of ten 30-s sleep epochs: the determination of a single sleep stage for the individual segment is complicated and shorter segments would not have been suitable for frequency-domain HRV analyses [7]. However, to prevent the bias caused by the wake periods, we excluded segments consisting of ≥50% wake.

A second limitation was only using a short-term period for HRV analysis instead of other analysis periods. To study the immediate effects of the desaturations on HRV, we selected short-term analyses owing to their suitability for this purpose [7]. Third, including patients with multiple comorbidities and medications in this study was a limitation because several comorbidities and anthropometric factors can affect HRV results alongside OSA (table 1) [37]. The results of the covariate-adjusted regression analyses showed that sex, BMI and history of arrhythmia were the confounding factors with the greatest effect on HRV findings alongside desaturation severity (table 5). However, the list of certain comorbidities and medications was incomplete and, thus, they were not used as exclusion criteria. Fourth, the desaturations and respiratory events were counted into the segment in which they started. The last desaturation or respiratory event of the 5-min segment could have continued to the next segment and so the immediate physiological response was not fully reflected in the segment in which it was counted into. A more thorough investigation is warranted that studies the simultaneous effect of the desaturations, respiratory events, sleep stages and other above-mentioned aspects on short-term HRV.

In conclusion, our results show that within a short-term period, the HRV of the patients with OSA suspicion increases with an increasing rate of respiratory events and severity of desaturations. These findings indicate that patients with suspected OSA with more severe desaturations demonstrate a shift towards sympathetic predominance, which in turn increases the risk for cardiovascular diseases. Moreover, ECG is routinely recorded as a part of PSG but it is not used in the current OSA diagnostics. With short-term HRV measurements, the immediate physiological consequences of OSA can be assessed, which cannot be done with long-term analyses. Therefore, a more detailed analysis of the ECG, HRV and desaturation characteristics could provide valuable information on the cardiovascular stress alongside the AHI when diagnosing OSA.
